# Supplementary material for: Prognostic peripheral blood biomarkers at ICU admission predict COVID-19 clinical outcomes
Source: Front Immunol. 2022 Nov 14;13:1010216. doi: 10.3389/fimmu.2022.1010216 (PMC9703061; doi:10.3389/fimmu.2022.1010216)
Supplement: Supplementary file 1 [file DataSheet_1.pdf]

Supplementary Material

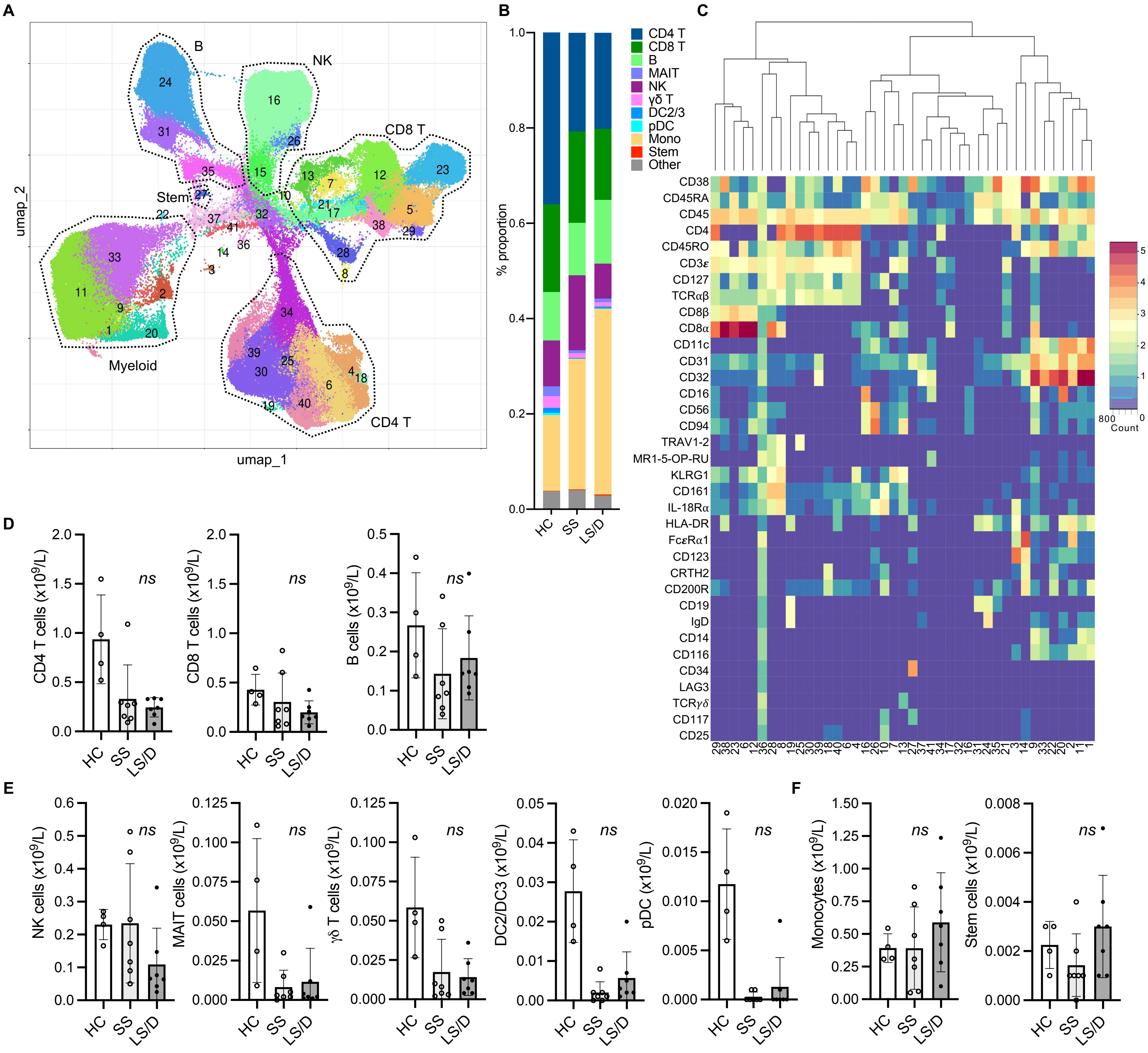
A
B
NK
CD8 T
Stem
Myeloid
CD4 T
umap_1
umap_2
B
% proportion
CD4 T
CD8 T
B
MAIT
NK
γδ T
DC2/3
pDC
Mono
Stem
Other
HC
SS
LS/D
C
CD38
CD45RA
CD45
CD4
CD45RO
CD3ε
CD127
TCRαβ
CD8β
CD8α
CD11c
CD31
CD32
CD16
CD56
CD94
TRAV1-2
MR1-5-OP-RU
KLRG1
CD161
IL-18Rα
HLA-DR
FcεRα1
CD123
CRTH2
CD200R
CD19
IgD
CD14
CD116
CD34
LAG3
TCRγδ
CD117
CD25
Value
Count
D
CD4 T cells (x10^9/L)
CD8 T cells (x10^9/L)
B cells (x10^9/L)
ns
E
NK cells (x10^9/L)
MAIT cells (x10^9/L)
γδ T cells (x10^9/L)
DC2/DC3 (x10^9/L)
pDC (x10^9/L)
F
Monocytes (x10^9/L)
Stem cells (x10^9/L)

Fig. S1. Major PBMC subsets fail to identify Long-Stay/Died patients (Initial Cohort). UMAP projection of ungated CyTOF-derived data from the Initial cohort (n=14) (**A**). Proportion of immune cell subsets in Healthy Controls (HC), Short-Stay (SS) and Long-Stay/Died (LS/D) patient outcome groups (**B**). Mean marker expression heatmap of clusters shown in a (**C**). Absolute counts of adaptive PBMC subsets (CD4 T, CD8 T, B) (**D**). Innate and unconventional subsets (NK, MAIT, γδ T, pDC, DC2/3) (**E**). Monocytes and stem cells (**F**). *, $p < 0.05$; **, $p < 0.01$; ***, $p < 0.001$; ns, $p \geq 0.05$ by two-tailed, two-sample unequal variance Student's t-test.

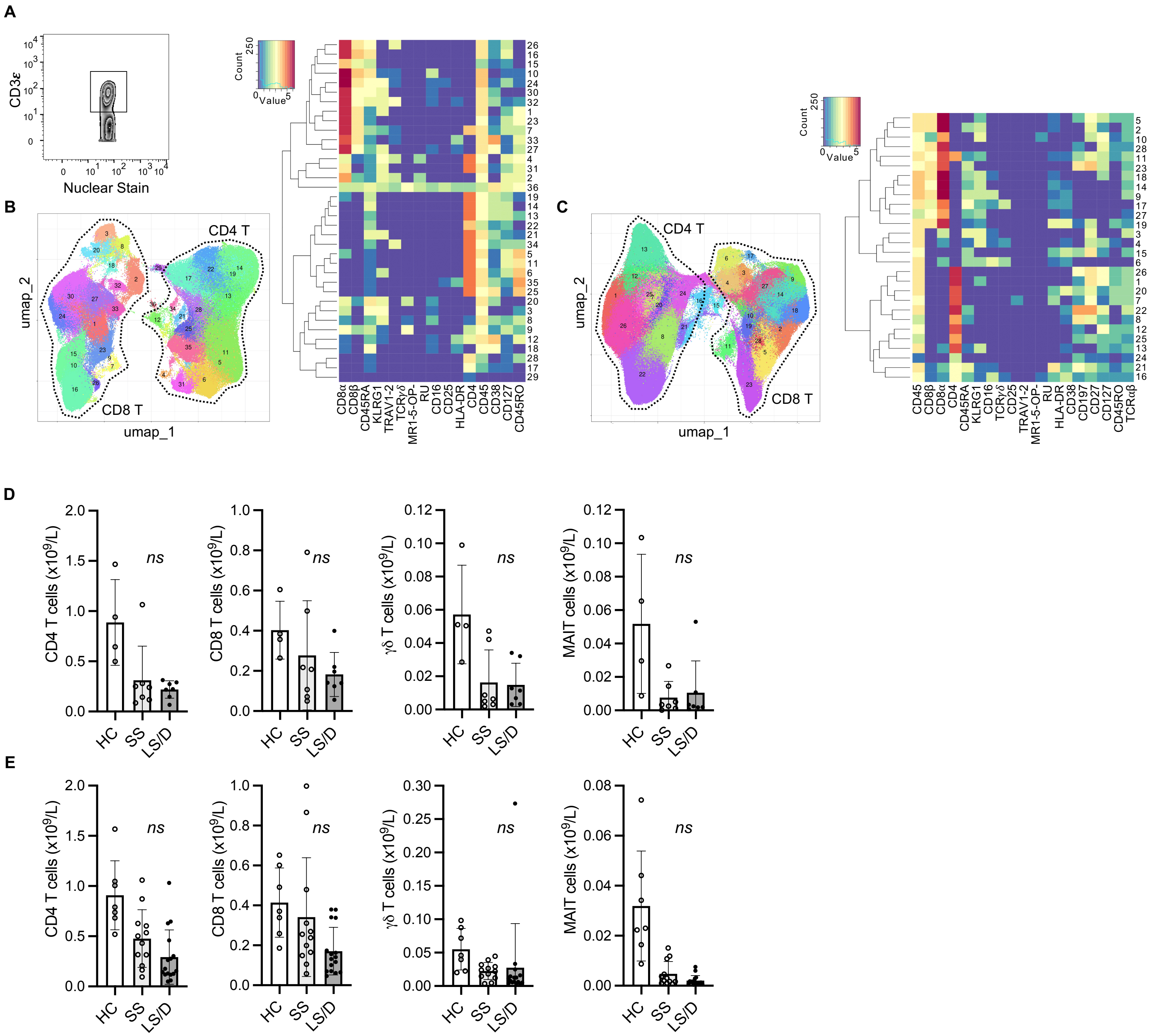

A
CD3ε
Nuclear Stain
Count
250
0
Value
5
CD8α
CD8β
CD45RA
KLRG1
TRAV1-2
TCRγδ
MR1-5-OP-RU
CD16
CD25
HLA-DR
CD4
CD45
CD38
CD127
CD45RO
B
CD4 T
CD8 T
umap_1
umap_2
C
CD4 T
CD8 T
umap_1
umap_2
CD45
CD8β
CD8α
CD4
CD45RA
KLRG1
CD16
TCRγδ
CD25
TRAV1-2
MR1-5-OP-RU
HLA-DR
CD38
CD197
CD27
CD127
CD45RO
TCRαβ
D
CD4 T cells (x10^9/L)
CD8 T cells (x10^9/L)
γδ T cells (x10^9/L)
MAIT cells (x10^9/L)
ns
HC
SS
LS/D
E
CD4 T cells (x10^9/L)
CD8 T cells (x10^9/L)
γδ T cells (x10^9/L)
MAIT cells (x10^9/L)
ns
HC
SS
LS/D

Fig. S2. T cell subsets fail to identify Long-Stay/Died patients. Representative gating of $CD3^+$ cells (**A**). Initial Cohort UMAP projections of $CD3^+$ gated cells (all samples combined; limited clustering channels) and mean marker expression heatmap (**B**). Same as in B but for the Replication Cohort (**C**). Initial Cohort absolute counts of T cell subsets identified based on gated clustering (**D**). **S**ame as in D but for the Replication Cohort (**E**). *, $p < 0.05$; **, $p < 0.01$; ***, $p < 0.001$; ns, $p \geq 0.05$ by two-tailed, two-sample unequal variance Student's t-test.

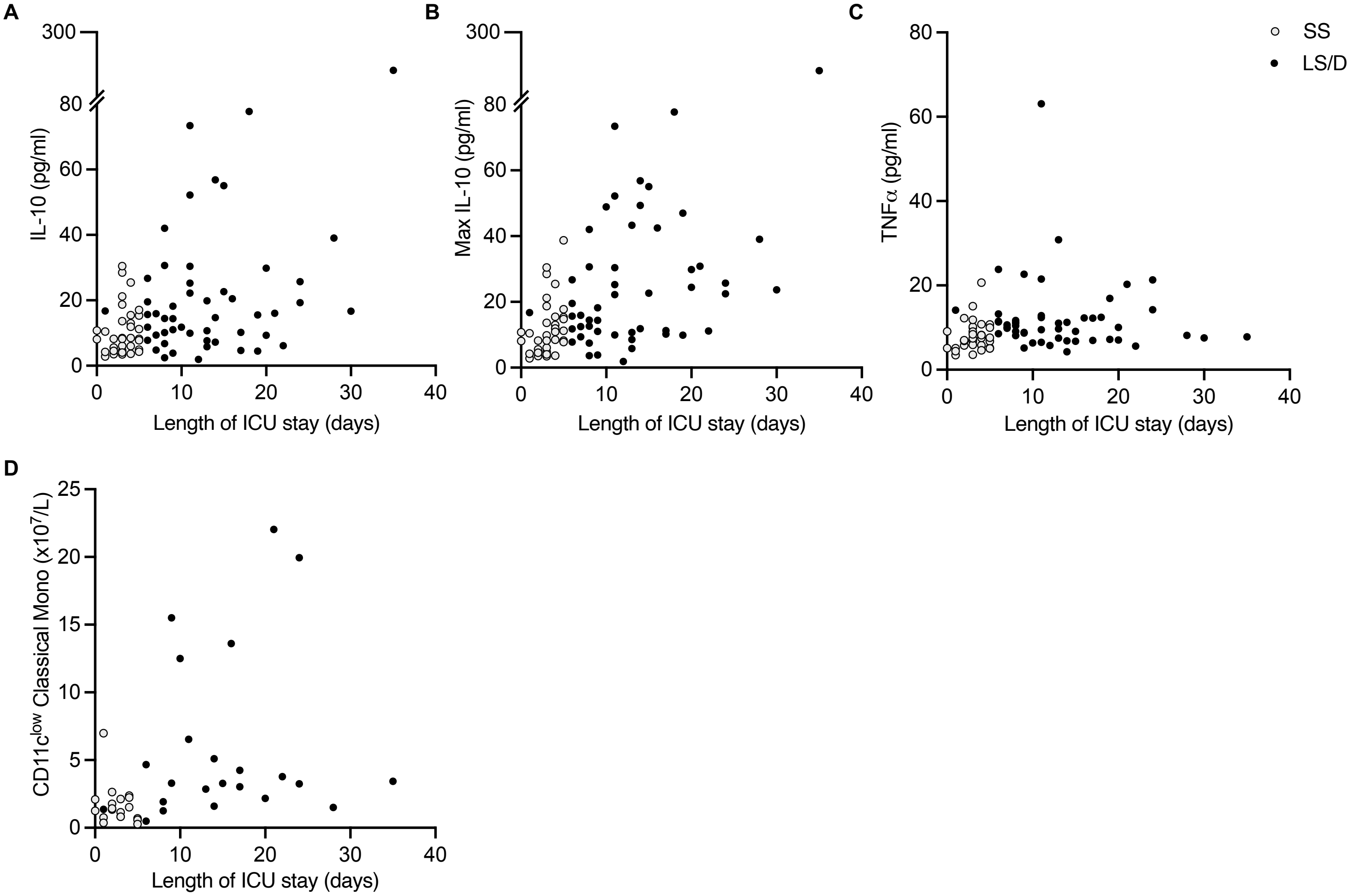
A
300
80
60
40
20
0
IL-10 (pg/ml)
0
10
20
30
40
Length of ICU stay (days)
B
Max IL-10 (pg/ml)
C
TNFα (pg/ml)
SS
LS/D
D
25
15
5
CD11c^low Classical Mono (x10^7/L)

Fig. S3. Cytokine and length of ICU stay correlations. Correlations of length of ICU stay with ICU admission serum IL-10 levels (**A**), maximum serum IL-10 during ICU stay (**B**) serum TNFα ICU admission levels (**C**) and CD11c^{low} Classical Monocytes (**D**). *, $p < 0.05$; **, $p < 0.01$; ***, $p < 0.001$; ns, $p \geq 0.05$ by two-tailed, two-sample unequal variance Student's t-test and R^2 by two-tailed Pearson correlation with 95% confidence interval.

Table S1. List of CyTOF antibodies

Antibody/Tetramer	Metal/Fluorophore*	Clone	Company	Used in Cohort
CD1c	151Eu	L161	**Biolegend**	Replication
CD3ε	143Nd	OKT3	**Biolegend**	both
CD4	174Yb	SK3	**Biolegend**	both
CD8α	168Er	SK1	**Biolegend**	both
CD8β	141Pr	SIDI8BEE	**eBioscience**	both
CD11c	147Sm	Bu15	**Biolegend**	both
CD14	153Eu	M5E2	**Biolegend**	both
CD16	158Gd	3G8	**Biolegend**	both
CD19	142Nd	HIB19	**Biolegend**	both
CD25	169Tm	BC96	**Biolegend**	both
CD27	175Lu	O323	**Biolegend**	Replication
CD31	145Nd	WM59	**Biolegend**	both
CD32	160Gd	IV.3	**Stemcell Technologies**	both
CD34	156Gd	581	**Biolegend**	both
CD38	106Cd	HIT2	**Biolegend**	both
CD45	89Y	HI30	**Biolegend**	both
CD45RA	110Cd	HI100	**Biolegend**	both
CD45RO	112Cd	UCHL1	**Biolegend**	both
CD56	148Nd	NCAM16.2	**BD Bioscience**	both
CD94 (NKG2C)	161Dy	DX22	**Biolegend**	both
CD116	150Nd	4H1	**Biolegend**	both
CD117	171Yb	104D2	**Biolegend**	Initial
CD123	164Dy	6H6	**Biolegend**	both
CD127	165Ho	A019D5	**Biolegend**	both
CD161	159Tb	HP-3G10	**Biolegend**	both
CD197/CCR7	171Dy	G043H7	**Biolegend**	Replication
CD200R	173Yb	0X-108	**BD Bioscience**	both
CD294 (CRTH2)	163Dy	BM16	**Biolegend**	both
CD301 (CLEC10A)	154Sm	H037G3	**Biolegend**	Replication
CD304 (NRP1)	172Yb	12C2	**Biolegend**	Replication
FcεRα1	176Yb	AER-37	**Biolegend**	both
HLA-DR	170Er	L243	**Biolegend**	both
IgD	116Cd	IA6-2	**Biolegend**	both
IL-18Rα	162Dy	H44	**Biolegend**	both
KLRG1	144Nd	SA231A2	**Biolegend**	both
LAG3	175Lu	11C3C65	**Biolegend**	Initial
TCRαβ	155Gd	T10B9.1A-31	**BD Bioscience**	both
TCR$\gamma\delta$	152Sm	B1	**Biolegend**	both
TRAV1-2	115Ln	3C10	**Biolegend**	both
Anti-APC (Secondary)	149Sm	APC003	**Biolegend**	both
MR1-5-OP-RU (Primary)	APC	NA	**NIH Tetramer Facility**	both

Table S2. Mean absolute counts and p-values (ungated cluster analyses)

Cohort	Total cell subset	H	SS	LS/D	H vs SS	H vs LS/D	SS vs LS/D
		Mean absolute counts (x10^9/L)			p-values		
Initial	CD4 T cells	0.936	0.330	0.244	0.067	0.053	0.549
	CD8 T cells	0.428	0.304	0.198	0.383	0.049	0.402
	B cells	0.267	0.143	0.184	0.176	0.335	0.509
	Monocytes	0.392	0.392	0.589	0.997	0.237	0.311
	Stem cells	0.002	0.001	0.003	0.247	0.315	0.088
	NK cells	0.230	0.234	0.109	0.961	0.032	0.150
	MAIT cells	0.057	0.008	0.012	0.122	0.140	0.721
	γδ T cells	0.058	0.018	0.014	0.077	0.065	0.705
	pDC	0.012	0.000	0.001	0.029	0.028	0.473
	DC2/3	0.028	0.002	0.006	0.026	0.034	0.205
Replication	CD4 T cells	0.911	0.511	0.309	0.008	<0.001	0.112
	CD8 T cells	0.386	0.374	0.184	0.914	0.005	0.090
	B cells	0.247	0.285	0.237	0.584	0.871	0.565
	Monocytes	0.403	0.657	0.740	0.041	0.028	0.618
	Stem cells	0.002	0.004	0.003	0.223	0.451	0.360
	NK cells	0.259	0.179	0.199	0.108	0.269	0.708
	MAIT cells	0.047	0.007	0.003	0.001	0.000	0.076
	γδ T cells	0.058	0.022	0.028	0.003	0.130	0.732
	pDC	0.009	0.001	0.001	<0.001	<0.001	0.575
	DC2/3	0.037	0.014	0.012	0.003	0.001	0.829

p-values calculated using a two-tailed, two-sample unequal variance Student's t-test.
H = Healthy; SS = Short-Stay; LS/D = Long-Stay/Died

Table S3. Mean absolute counts and p-values (gated monocyte and T cell cluster analyses)

Cohort	Monocyte subset	H	SS	LS/D	H vs SS	H vs LS/D	SS vs LS/D
		Mean absolute counts ($x10^9/L$)			p-values		
Initial	CD11c^{low} Classical	0.004	0.019	0.082	0.536*	0.133*	0.388*
	Total Classical	0.108	0.110	0.181	0.620	0.136	0.212
	Total Intermediate	0.218	0.247	0.377	0.939	0.234	0.338
	Total Non-Classical	0.019	0.020	0.011	0.844	0.080	0.462
	CD4 T cells	0.888	0.312	0.219	0.010	0.004	0.506
	CD8 T cells	0.403	0.277	0.182	0.309	0.005	0.415
	MAIT cells	0.052	0.008	0.011	0.014	0.018	0.724
	γδ T cells	0.057	0.016	0.015	0.009	0.006	0.868
Replication	CD11c^{low} classical	0.001	0.015	0.058	<0.001*	0.010*	0.076*
	Total Classical	0.117	0.193	0.271	0.035	0.010	0.167
	Total Intermediate	0.196	0.412	0.422	0.025	0.021	0.925
	Total Non-Classical	0.022	0.026	0.015	0.890	0.061	0.404
	CD4 T cells	0.899	0.477	0.292	0.004	0.061	0.098
	CD8 T cells	0.412	0.341	0.171	0.489	<0.001	0.083
	MAIT cells	0.035	0.005	0.002	0.001	<0.001	0.093
	γδ T cells	0.055	0.022	0.028	0.003	0.140	0.769

p-values calculated using a two-tailed, two-sample unequal variance Student's t-test.
H = Healthy; SS = Short-Stay; LS/D = Long-Stay/Died
*p-values that have been Bonferroni-adjusted for multiple comparisons

Table S4. Cell surface protein signatures for cluster assignment

Analyses	Cell type	Cell surface protein signatures for cluster assignment
Ungated	CD4 T cells	CD3+ TCRαβ+ CD4+ CD8-
	CD8 T cells	CD3+ TCRαβ+ CD4- CD8+
	B cells	HLA-DR+ IgD+ CD19+
	Monocytes	CD116+ CD3- CD19- CD11c+ HLA-DR+ CD32+
	Stem cells	CD34+
	NK cells	CD94+ CD3- CD116-
	MAIT cells	CD3+ TCRαβ+ IL-18Rα+ MR1-5-OP-RU+
	γδ T cells	CD3+ TCRαβ- TCRγδ+
	pDC	CD11c- NRP1+ CD123+ FcεRα1+ CD116+ HLA-DR+
	DC2/3	CD11c+ CD1c+ NRP1- CD123+ FcεRα1+ HLA-DR+
Gated on CD3- CD19- CD116+ (Monocytes)	CD11c^{low} classical	CD116+ CD14^{high} CD16- CD11c^{low}
	Total Classical	CD116+ HLA-DR+ CD14^{high} CD16- CD123-
	Total Intermediate	CD116+ HLA-DR+ CD14$^{high/int}$ CD16^{low} CD123^{low}
	Total Non-Classical	CD116+ HLA-DR+ CD14^{low} CD16^{high}

Table S5. Sensitivity and specificity calculations for serum IL-6.

	Sensitivity	Specificity	False Positives	False Negatives	LR+
IL-6 screen (Cytokine Cohort; n = 90)	39% (22/56)	76% (22/29)	24% (7/29)	42% (24/56)	1.6
